# Formation of Chlorine in the Atmosphere by Reaction of Hypochlorous Acid with Seawater

**DOI:** 10.1021/acs.jpclett.3c03035

**Published:** 2024-01-08

**Authors:** Imon Mandal, Natalia V. Karimova, Itai Zakai, R. Benny Gerber

**Affiliations:** † The Fritz Haber Center for Molecular Dynamics, Institute of Chemistry, The Hebrew University of Jerusalem, Jerusalem 91904, Israel; ‡ Department of Chemistry, University of California, Irvine, California 92697, United States

## Abstract

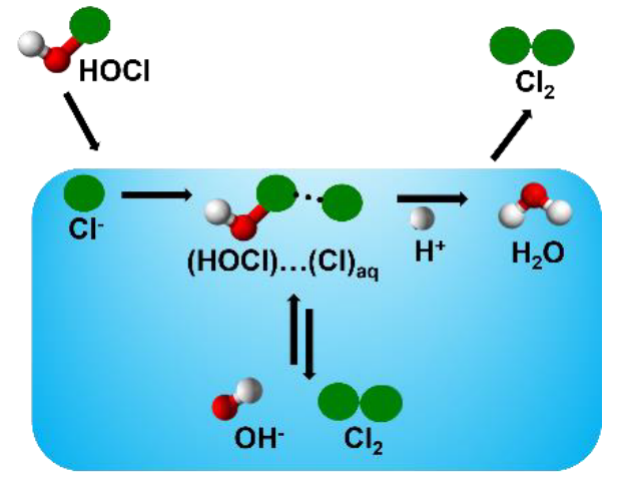

The highly reactive dihalogens play a significant role in the oxidative chemistry of the troposphere. One of the main reservoirs of these halogens is hypohalous acids, HOX, which produce dihalogens in the presence of halides (Y^–^), where X, Y = Cl, Br, I. These reactions occur in and on aerosol particles and seawater surfaces and have been studied experimentally and by field observations. However, the mechanisms of these atmospheric reactions are still unknown. Here, we establish the atomistic mechanism of HOCl + Cl^–^ → Cl_2_ + OH^–^ at the surface of the water slab by performing ab initio molecular dynamics (AIMD) simulations. Main findings are (1) This reaction proceeds by halogen-bonded complexes of (HOCl)···(Cl^–^)_aq_ surrounded with the neighboring water molecules. (2) The halogen bonded (HOCl)···(Cl^–^)_aq_ complexes undergo charge transfer from Cl^–^ to OH^–^ to form transient Cl_2_ at neutral pH. (3) The addition of a proton to one proximal water greatly facilitates the Cl_2_ formation, which explains the enhanced rate at low pH.

Reactive halogen species (RHS such as X, XY, and XO, where X, Y = Cl, Br, or I) play an important role in the chemistry and oxidizing capacity of the troposphere as well as stratosphere.^1−7^ Firstly, the RHS act as an effective sink for ozone (O_3_) by depleting O_3_ through efficient catalytic cycles. Second, they influence the nitrogen oxides (NO_*x*_), and HO_*x*_ cycles.^4−6^ RHS also impact the lifetimes of reduced trace gases such as methane, non-methane volatile organic compounds, and dimethyl sulfide, as well as mercury in the atmosphere.^1,4,8^ These RHS originate from different sources, including organohalogen oxidation,^1^ ozone deposition to the ocean surface,^9^ and release from sea salt aerosols.^5,7,10^ The release occurs via the uptake of hypohalous acid species (HOX, where X is equal to Br, Cl, or I) from the gas phase^11^ or hydrolysis of N_2_O_5_ forming ClNO_2_^12^ or hydrolysis of XNO_3_ forming HOX.^6^ However, alternation of atmospheric acidity due to changes in acid precursor gases emissions influence the RHS formation.^13^ In fact, halogen formation gets promoted by the acid-driven reactions^14−16^
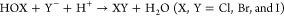
R1In these reactions, the protons (H^+^) are incorporated into the reaction products formed, leading to acid-driven reactions. Sea salt aerosols originate at the same pH as seawater (∼8) but, within minutes, undergo a pH drop of a few pH units.^17^ However, the value of pH ∼ 8 is an average value, and there are many conditions where locally seawater is acidic. The roles of seawater, aerosols, and the acidity of those media in halogen activation in the atmosphere, however, remain poorly understood and need to be addressed to further advance our understanding of the complex role of acidity in the atmosphere.

Among halogens, Cl contributes for 5.4–11.6% of total methane sinks and involved to a lesser extent for ozone destruction.^18^ Additionally, chloride (Cl^–^) is the greatest abundant halide anions in seawater, aerosol and most aqueous systems in the atmosphere.^4^ However, the mechanisms responsible for the oxidation of Cl^–^ to reactive forms (e.g., Cl^•^, ClO, Cl_2_, BrCl, HOCl, and ClNO_2_) are incompletely investigated.^19^ Consequently, assessing the impacts of reactive chlorine on atmospheric chemistry become challenging. On the other hand, Cl is also used globally as chemical oxidant for drinking water disinfection due to its cost effectiveness. During water treatment, hypochlorous acid (HOCl) acts as the major reactive form among the different seawater like Cl species and most of the elementary oxidation of halides (Y^–^ where Y = Cl, Br, and I) reactions start with HOCl.^20^ Another important fate of HOCl in the atmosphere is the heterogeneous reaction with halide anions in or on condensed aqueous phases, such as sea salt aerosol particle surfaces or seawater surfaces (R1) forming Cl_2_ followed by photolysis generation of RHS Cl radicals. This heterogeneous reaction becomes faster in acidic environment as found in the laboratory experiments^21^ and recently observed in field studies.^19,22^ Despite its importance, the atomistic details for these atmospheric important reactions with water medium have not been established until date. Thus, in this communication we aim to investigate the chemical pathway and role of water medium for HOCl reacting with the most abundant halide Cl^–^, providing valuable insights about the formation of dihalogen Cl_2_ in the atmosphere.

We develop a minimalistic computational model with (HOCl)···(Cl^–^)_aq_ complex and water slab containing 72 water molecules^23−25^ to gain microscopic insight into the structures of the pre-reactive complexes, their lifetimes and the mechanisms of the reaction.^26^ There are two possible orientations for the HOCl and Cl^–^ molecules to form the pre-reactive complexes: (a) the hydrogen bonded complex, in which the HOCl donates a hydrogen bond to Cl^–^ (b) the halogen bonded complex, in which the Cl^–^ acts as a nucleophile to the slightly electrophilic area on the Cl atom of HOCl. Our model unravels that halogen bonded complexes of the (HOCl)···(Cl^–^)_aq_ system play the major role in the dihalogen formation. The importance of halogen bonded complexes have also been reported for the halogen exchange reaction in (HOCl)···(I^–^)_aq_ and were explored spectroscopically.^27^ Here, with AIMD simulations, we report the microscopic details of Cl_2_ formation at acidic pH. Interestingly, under neutral pH condition, although Cl_2_ forms transiently, it was not released in the subsequent process.

*Stability of (HOCl)···(Cl−)_aq_ Complexes in Water.* The reaction HOCl + Cl^–^ → Cl_2_ + OH^–^ in/on aerosol or seawater is not expected to proceed in a ballistic way but through a formation of pre-reactive complex of (HOCl)···(Cl^–^)_aq_. Gas phase calculations suggest that two possible structures are feasible for the HOCl and Cl^–^ molecules to form the pre-reactive complexes: (a) the hydrogen bonded complexes^28^ and (b) the halogen bonded complexes.^27^ As the pre-reactive complexes allow the two reagents HOCl and Cl^–^ to interact before they react, the stability and lifetimes of these pre-reactive complexes greatly affect the reaction mechanism. To study the complexes in water, we have employed an ab initio model of liquid water slab with periodic boundary conditions, as described in detail in the Computational Methods section (*vide infra*). We substitute hydrogen atoms with deuterium to accommodate a larger time step for the simulations. We first calculate the gas phase optimized structure (geometric parameters of these structures are provided in Table S1), position on the water slab model and simulate the system for 20 ps (ps). All the simulations resulted in the formation of the halogen and hydrogen bonded (HOCl)···(Cl^–^)_aq_ pre-reactive complexes (Figures 1, S1, and S2). Figures 1b, S1, and S2 show the root mean square deviation (RMSD) changes, which is an overall average measure to track the evolution of the complex structure along an MD trajectory with respect to a reference structure (here gas phase structure). These figures indicate two significant results: (a) both the halogen and hydrogen bonded (HOCl)···(Cl^–^)_aq_ complexes do not dissociate in water for at least 20 ps (our simulation time). This stability does not pertain to the trajectories where reaction occurs. In transiently reacting trajectories, the complexes undergo temporary reactions and return to the complex form. (b) (HOCl)···(Cl^–^)_aq_ complexes are similar to the initial gas phase structure. The stability against dissociation is remarkable for hydrogen bonded complexes. However, the fluctuations in RMSD for the hydrogen bonded (HOCl)···(Cl^–^)_aq_ complexes are up to 3 Å (1 Å = 1× 10^–10^ m) due to the competition of hydrogen bonding partner of HOCl between Cl^–^ and surrounding waters (Figure S2). Fluctuation of halogen bonded (HOCl)···(Cl^–^)_aq_ complexes are ∼1 Å except one trajectory where the HOCl dissociates from the complex (Figure S1). This stabilized water envelop provides enough lifetime to the halogen bonded complexes. We implemented geometrical criteria to determine the formation of hydrogen and halogen bonds:^29,30^ a hydrogen bond is defined by an H–Cl^–^ distance smaller than 3.2 Å, and an ∠O–H···Cl^–^ angle larger than 140°. A halogen bond is defined by a Cl–Cl^–^ distance smaller than 3.5 Å and a ∠Cl^–^...Cl–O angle which is larger than 130°. Although, the gas phase calculations reveal that hydrogen bonded (HOCl)···(Cl^–^) is 7.6 kcal/mol (1 kcal/mol = 4.184 kJ/mol) more stable than the halogen bonded (HOCl)···(Cl^–^) complex, in the presence of water both hydrogen and halogen bonded (HOCl)···(Cl^–^)_aq_ complexes exhibit a minimum lifetime of 20 ps. While, hydrogen bonded pre-reactive complexes have been recognized as significant players in the reaction mechanisms,^31,32^ in this study, we shed light on the crucial role of halogen bonded (HOCl)···(Cl^–^)_aq_ complexes in the formation of atmospheric Cl_2_. Interestingly, hydrogen bonded complexes in all 5 simulations remain unreactive (Figure S3). Our simulations provide compelling evidence that halogen bonded (HOCl)···(Cl^–^)_aq_ complexes persist for a sufficient duration in water, serving as crucial precursors to the reaction.

**Figure 1:**
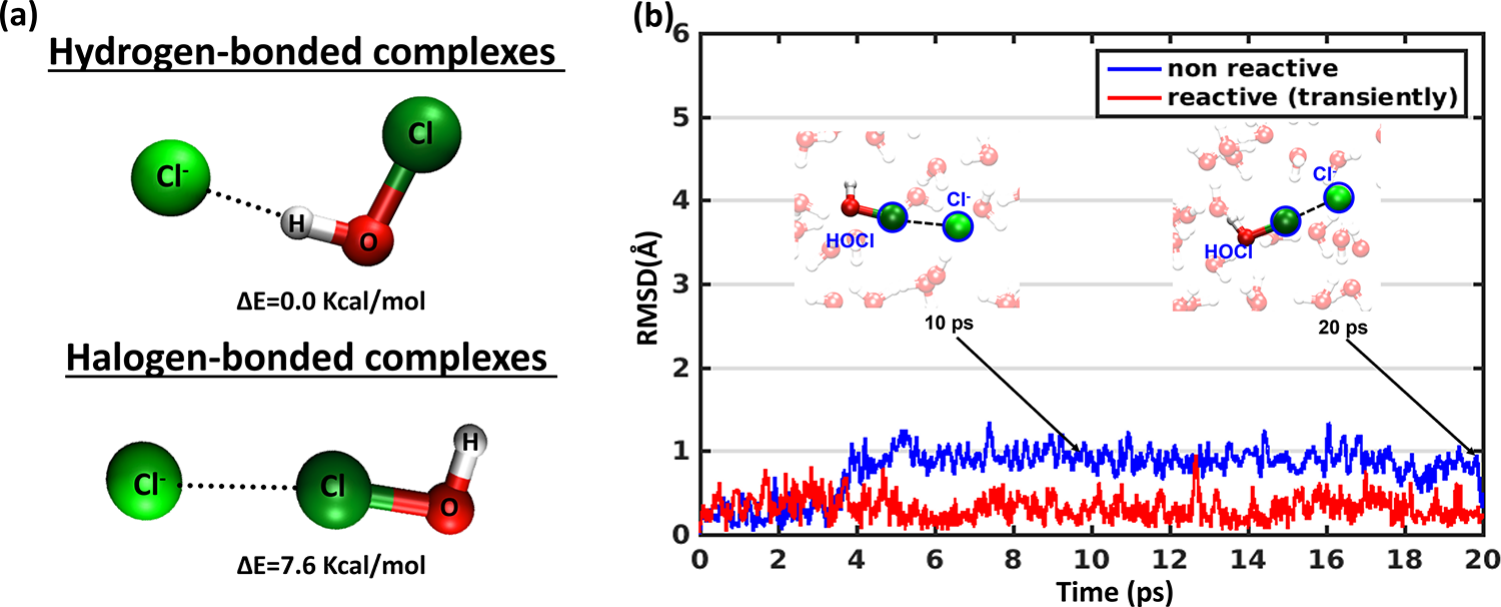
(a) Gas phase optimized structure (B3LYP/def2-TZVPD) of the hydrogen and halogen bonded pre-reactive complexes of (HOCl)···(Cl^–^). (b) RMSD of the halogen bonded (HOCl)···(Cl^–^)_aq_ complexes (4 atoms H, O, Cl and Cl^–^) along the time trajectory shows the complexes are stable at least 20 ps. Non-reactive legend indicate one representative trajectory among 7 trajectories which remain unreactive during simulations. Reactive demonstrates data from one representative trajectory among 3 trajectories which show transient reactions depicted in next section. Snapshots of the halogen bonded (HOCl)···(Cl^–^)_aq_ complexes at time 10 and 20 ps for representative unreactive simulation are shown in the inset.

*Reaction of HOCl with Seawater Yield Transient Cl_2_ Formation at Neutral pH.* Although the overall RMSD demonstrate the stability of the halogen bonded (HOCl)···(Cl^–^)_aq_ complexes, time evolution of pairwise bond lengths of Cl–Cl^–^ and Cl–O indicate transient formation of Cl_2_ formation in 3 simulations at neutral pH. Figure 2a shows the time evolution of the bond lengths related to the Cl_2_ formation reaction in one of the 3 transiently reactive simulations. The Cl–Cl^–^ bond length decreases to 2.3 Å (blue) and the Cl–O bond length increases (red) at the same time (region between black lines) indicating the Cl_2_ formation. The Hirshfeld partial charge analysis^33^ also reveals the similar transition in the same time region around 5 ps (region between black lines). The partial charge on the Cl^–^ (light green) ion was transferred to OH (red). Thus, the partial charge on Cl^–^ increases, decreasing the charge of OH. Then, after 1 ps the Cl_2_ falls apart and forms back halogen bonded (HOCl)···(Cl^–^)_aq_ complex. Data from the other 2 transiently reactive simulations are provided in Figure S4. The transient reaction times vary from few 100s of femtoseconds (fs) to 2 ps. Hence, these findings suggest that the reaction conditions that stabilize the charge transferred to OH would facilitate the Cl_2_ formation. This leads to the next section of the study depicting the effect of addition of proton on the reaction.

**Figure 2:**
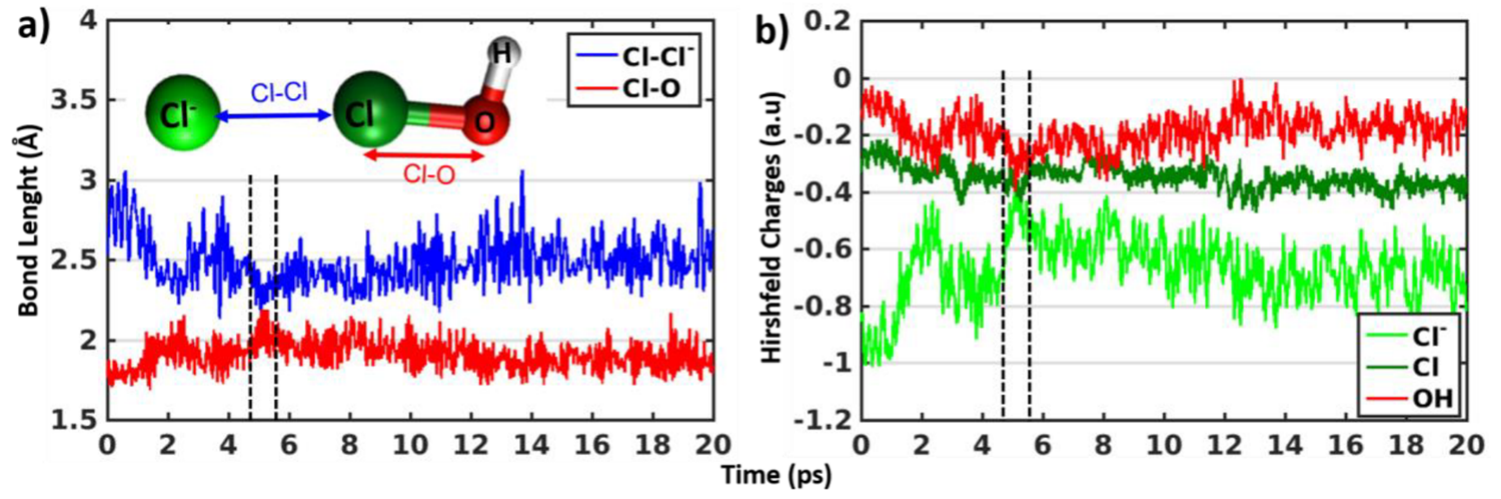
(a and b) Time evolution of the bond lengths (Cl–Cl^–^ and Cl–O) and Hirshfeld partial charges (Cl^–^, Cl, and summation of O and H from HOCl) of halogen bonded (HOCl)···(Cl^–^)_aq_ complexes along trajectory. The black dotted lines in parts a and b are eye guides for the time of the transient Cl_2_ formation at neutral pH. Structure of the halogen bonded (HOCl)···(Cl^–^)_aq_ complexes (4 atoms H, O, Cl and Cl^–^) with color coded bond lengths (for a) and atoms (for b) are provided in the inset of part a.

We investigated the HOCl + Cl^–^ → Cl_2_ + OH^–^ also with small water cluster model of 6 water molecules. Water cluster results indicate that the pre-reactive halogen bonded complexes are thermodynamically more stable than the product state. The calculations were carried out by using the MP2/6-311++G**//PBE0-D/6-31+G* method (Figure S5). The results revealed a mechanism where the formation of a Cl_2_ molecule occurs through a proton transfer process from one of the water molecules to a hydroxide fragment in the HOCl molecule. Interestingly, the stability of the post-reaction complex was observed only under specific conditions, where the Cl_2_ molecule and OH^–^ ion were separated by multiple water molecules. In contrast, when the separation distance was insufficient, the formation of the Cl_2_ product molecule could not be achieved.

*Effect of Proton (Low pH) on the Cl_2_ Formation Reaction.* Chemical reactions influenced by acidity have a significant effect on the tropospheric multiphase oxidant budget. Here, we investigate the effect of acidity on the Cl_2_ formation reaction and mimic the acidity or low pH of the water medium by adding a proton to a neighboring water molecule forming hydronium ion. Our simulations with hydronium ion indicate that HOCl + Cl^–^ + H^+^ → Cl_2_ + H_2_O reaction proceeds via a mechanism where the proton reacts with halogen bonded (HOCl)···(Cl^–^)_aq_ complex. Figure 3c shows the time evolution of the bond lengths related to the Cl_2_ and H_2_O formation reaction. The Cl–Cl^–^ bond length decreases to 2.0 Å (blue) and the Cl–O bond length increases (red) at the same time (black line) indicating the Cl_2_ formation. The Hirshfeld partial charge analysis also reveals a similar transition in the same time region around 100 fs (black line). The partial charge on the Cl^–^ (light green) ion got transferred to OH (red) and form neutral H_2_O (Figure 3d). The O–H bond length (green in Figure 3c), which depicts the bond distance between the O atom of HOCl moiety and one H of the hydronium ion, also indicates the formation of H_2_O. Data from the whole ∼20 ps simulation (Figure S6 and Figure 4) shows that Cl_2_ formation is nonreversible and will not revert to a halogen bonded (HOCl)···(Cl^–^)_aq_ complex. The time evolution of the distance between the geometric center of the water box and the geometric center of the Cl_2_ formed (Figure 4a) reveals that after 20 ps Cl_2_ would detach from the slab. The Hirshfeld partial charge analysis also suggests that Cl_2_ loses all interactions with all neighboring atoms leading to complete neutral partial charge for Cl_2_ (Figure 4b). Data pertaining to simulations resulting nonreversible Cl_2_ are provided in Figure S7 and simulation leading to the detachment of Cl_2_ can be found in Figure S8. The enhancement of the heterogeneous Cl_2_ formation reaction in acidic environment collaborated well with laboratory experiments^21^ and field observations.^19,22^

**Figure 3:**
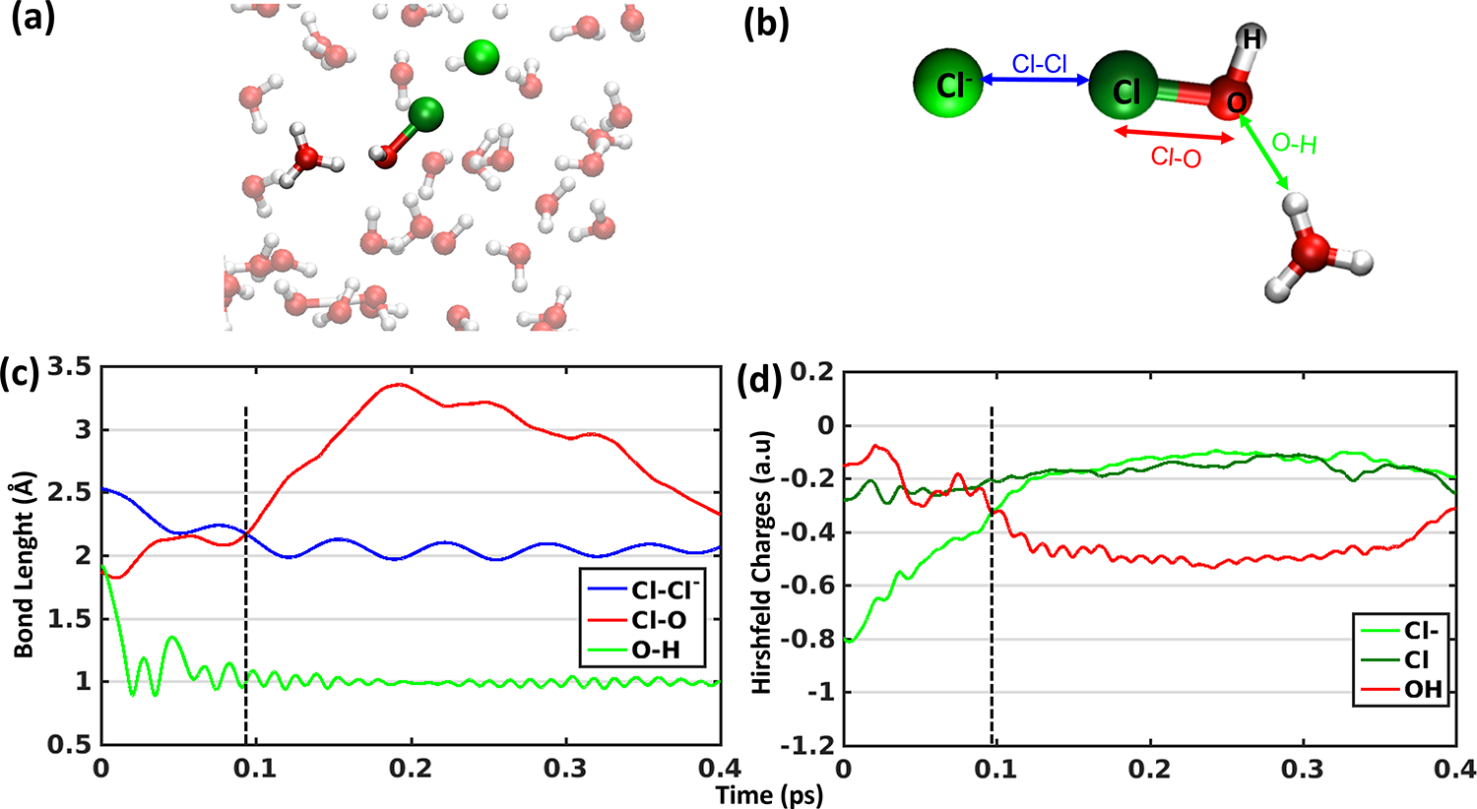
(a) Starting structure of halogen bonded (HOCl)···(Cl^–^)_aq_ complexes with the extra proton added on a neighboring water molecule forming hydronium ion. (b) Enlarged view of 4 atoms H, O, Cl and Cl^–^ of (HOCl)···(Cl^–^)_aq_ complexes and the hydronium ion with color coded bond lengths (for c) and atoms (for d). (c and d) Time evolution of the bond lengths (Cl–Cl^–^, Cl–O and O–H) and Hirshfeld partial charges (Cl^–^, Cl and summation of O and H from HOCl) of halogen bonded (HOCl)···(Cl^–^)_aq_ complexes along trajectory. The black dotted lines in parts c and d are eye guides for the time of the non-reversible Cl_2_ formation at acidic pH. Data for whole simulation are available in Figure S6.

**Figure 4:**
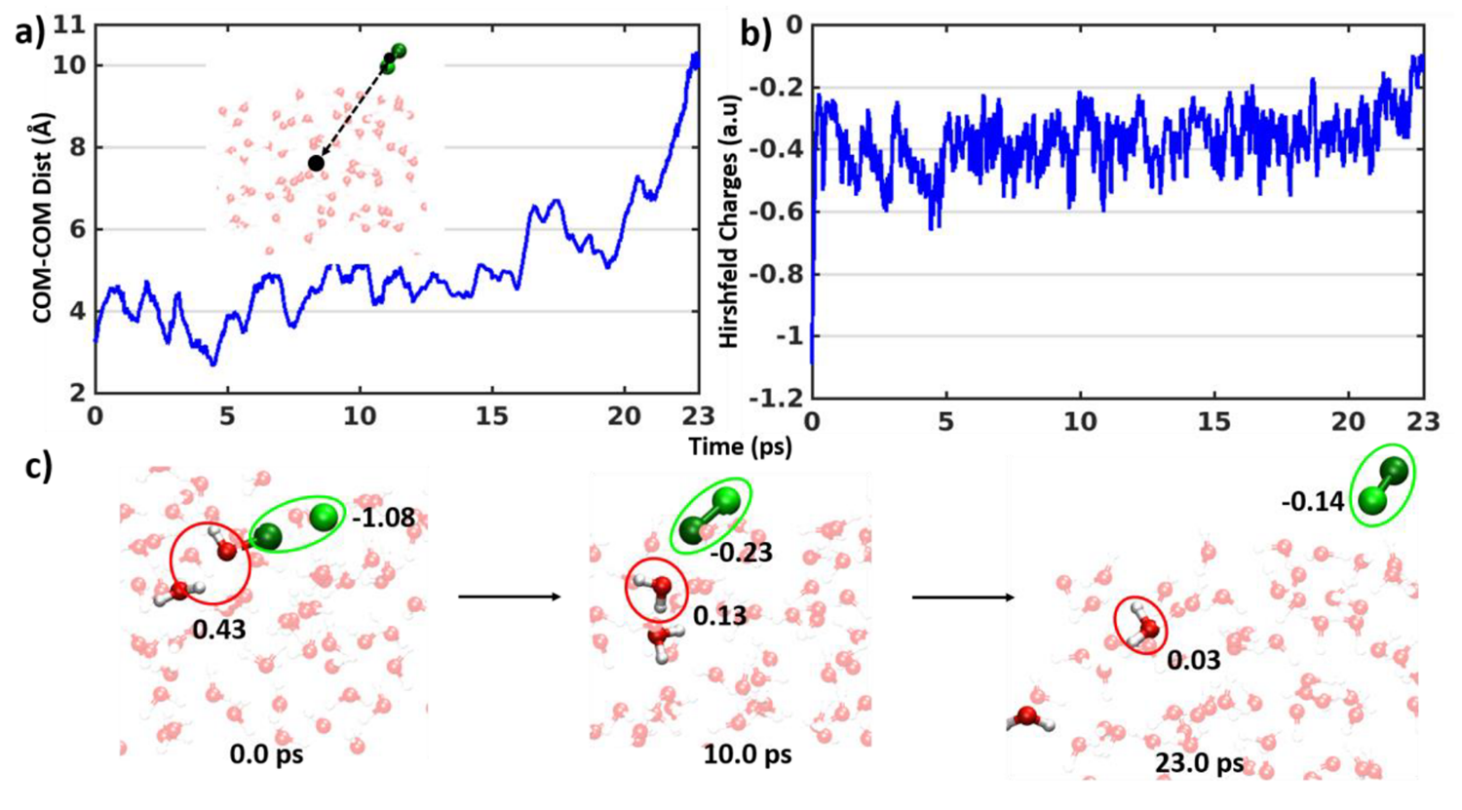
(a and b) Time evolution of the distance between geometric center of the water slab and the formed Cl_2_ (*inset*) and Hirshfeld partial charge (summation of Cl^–^ and Cl from HOCl) of halogen bonded (HOCl)···(Cl^–^)_aq_ complexes along trajectory. (c) Snapshots of the halogen bonded (HOCl)···(Cl^–^)_aq_ complexes with hydronium ion at different time steps with the Hirshfeld charges of Cl_2_ and H_2_O are mentioned on those.

As during the Cl_2_ formation in acidic medium, the extra proton departs from the hydronium ion and effectively attaches to OH forming a H_2_O (water), the correct alignment of one of the protons of the hydronium ion to OH of HOCl is crucial. 2D potential energy scan (Figure 5) along the distance (*r*) between the closest proton of OH to the O atom and angle to be formed between OH and proton (*θ*) shows a minimum suggesting preorganization of hydronium ion. This result indicates that one of the protons of the hydronium ion must be within a distance of less than 2 Å from the transiently generated hydroxide ion. Moreover, the Cl_2_ formation highly depends on the specific orientation of the hydronium ion, and the θ angle to be formed during the process requires to be ∼80–120° angle (Figure 5). These findings align with previous research for bromide radical formation^34^ and proton transfer process in water medium.^35^ These studies demonstrated the significant role of relative orientations of the reactants in facilitating their entrance into the “cone of acceptance”, which is compatible with the formation of the specific HOH angle of the product water molecule. In our simulations also, we observe that when the dynamics is started with θ ∼ 80–120° (Table S2) that leading to the formation of the bond angle of water (∼104°), the system transitions to products in less than 300 fs (Figure 3 and Figure S7). Other θ orientations are not exhibiting any reaction in the simulation time scale.

**Figure 5:**
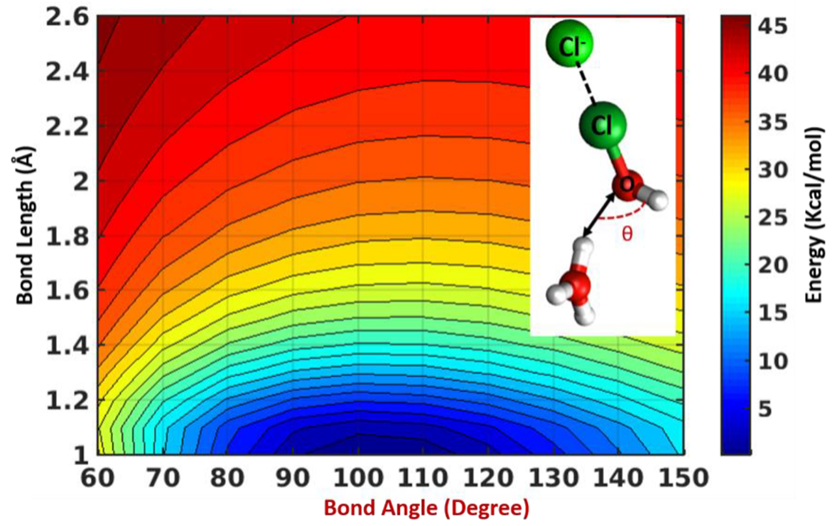
2D potential energy scan along bond length between O atom of HOCl (*r*) and proton from hydronium ion and θ (description in text) for halogen bonded (HOCl)···(Cl^–^)_aq_ complexes in acidic pH.

In this study, we establish the atomistic mechanism of the HOCl + Cl^–^ → Cl_2_ + OH^–^ reaction at the air–water interface. Initially, the reaction proceeds by the formation of pre-reactive complexes of (HOCl)···(Cl^–^)_aq_ surrounded with the neighboring water molecules. Both the hydrogen bonded complex and the halogen bonded complex are thoroughly investigated in the study. Our simulations reveal the significant role of the halogen bonded (HOCl)···(Cl^–^)_aq_ complex in the process of atmospheric Cl_2_ formation. The substantial lifetime of 20 ps, for the halogen bonded complexes in water at neutral pH suggests that their role as reaction precursors in the chemistry of aqueous environments may be more critical than conventionally assumed. The Cl_2_ formation reaction proceeds by halogen bonded complexes of (HOCl)···(Cl^–^)_aq_ surrounded with the neighboring water molecules and undergo charge transfer from Cl^–^ to OH^–^ to form transient Cl_2_ at neutral pH. Addition of a proton to one proximal water greatly facilitates the Cl_2_ formation which explains the enhanced experimental rate at low pH. Indeed, this proton addition mimicking the low pH experimental condition stabilizes the charge on OH^–^ forming neutral H_2_O (water). Furthermore, the specific orientation of the nearest proton of hydronium ion to OH of HOCl is essential for the reaction to proceed. Moreover, because of the increased entropy effect due to water formation, the reaction is expected to be enhanced at the interface with respect to bulk. On the other hand, despite the sufficient lifetime of the hydrogen bonded (HOCl)···(Cl^–^)_aq_ complexes in water, those remain unreactive throughout simulations. In a parallel study on halogen bonded complexes of (HOCl)···(I^–^)_aq_ from our lab establish the pivotal role of halogen bonded complexes in the halogen exchange reaction HOCl + I^–^ → HOI + Cl^–^. This study also highlights the enormous enhancement in the reaction rate compared to that in the gas phase reaction due to the catalytic effect of the waters from the slab. In summary, our study suggests the desirability of future investigations of the potential role of halogen-bonded complexes of halogen-containing molecules as precursors or intermediates in various atmospheric reactions at air–water interfaces or in a water medium. These studies also unveil some atmospheric implications such as (1) at acidic aqueous environments (aerosol, seawater) dihalogen formation would be dominant, whereas at neutral pH halogen exchange would play a significant role. This would have substantial consequences for the dihalogen and hypohalous acid distribution in the atmosphere.

## Computational Methods

*DFT Calculations:Gas-phase optimization and 2D Potential Energy Scan.* Minimum energy structures of (HOCl)···(Cl^–^) complex in gas phase are calculated using method B3LYP^36^ and def2-TZVPD basis.^37^ We perform a 2D potential energy scan along the distance between the closest proton of OH to O atom and angle to be formed between OH and proton (*θ*) using PBE exchange correlation functional^38^ and cc-PVDZ basis set. All the single point calculations with opt = modred option are implemented in Gaussian 16, Revision C.01.^39^

*Ab Initio Molecular Dynamics (AIMD) Simulations with Water Slab.* We adopt similar procedures for our simulations utilized by the Gerber group in several previous publications^23,25^ to describe chemical reactions on water slab. The unit cell of the slab is modeled by 72 water molecules in a 13.47 × 15.56 × 40 Å^3^ rectangular box. Periodic boundary conditions are employed in *x* and *y* but not in the *z* direction, mimicking the water surface in the *xy* plane. A water molecule at the center of the box is replaced with the gas phase optimized (HOCl)···(Cl^–^) complex. The system is then equilibrated at 300 K for ∼3.0 ps using a Nosé–Hoover massive thermostat,^40^ with a 0.5 fs time step until the (HOCl)···(Cl^–^) complex reaches to the topmost layer of the water slab. Subsequently, these systems are simulated for 20 ps, resulting in the formation of the halogen and hydrogen bonded (HOCl)···(Cl^–^)_aq_ complexes. From the second step 10 simulations for halogen bonded (HOCl)···(Cl^–^)_aq_ complexes and 5 simulations for hydrogen bonded (HOCl)···(Cl^–^)_aq_ complexes are performed for 20 ps initializing with different velocities using different SEED parameter in CP2K 7.1.^41^ Similarly, we introduce one extra proton to neighboring water forming hydronium ion for the simulations mimicking acidic pH. The protons are incorporated to the water in the structures derived from the neutral pH simulations, where one proton of water is in a specific angle orientation as discussed above (*vide supra*). Considering the fast nature of proton transfer from hydronium to OH of HOCl, we employ 0.2 fs time step for first 4 ps. Subsequently, the time step is increased to 0.5 fs for the rest of the simulations.

All ab initio molecular dynamics simulations are performed using the QUICKSTEP module^42^ of CP2K 7.1^41^ employing Perdew–Burke–Ernzerhof density functional^38^ with a Grimme dispersion correction (PBE-D3).^43,44^ Previous study from our group with this DFT potential for this type of charge transfer reactions supports the reliability in this study.^23^ Double-zeta valence polarization basis-set (DZVP-MOLOPT-SR)^45^ and the Goedecker–Teter–Hutter (GTH) pseudopotentials^46^ are employed for the computations. We treat the long-range electrostatic interactions using the Martyna–Tuckerman algorithm.^47^ For the plane-wave basis set, a cutoff of 320 Ry (1 Ry = 1.097 × 10^7^ m^−1^) is employed, along with a relative cutoff of 50 Ry. All hydrogen atoms are substituted by deuterium to accommodate a larger time step of 0.5 fs for simulations. This choice is substantiated, as nuclear quantum effects are not anticipated to have a substantial impact on the properties detailed in this section.

In our analysis, we examine the Hirshfeld charges^33^ generated from CP2K 7.1.^41^ This charge analysis is based on the concept of describing the molecule by dividing it into its constituent atoms and gauging how these atoms deviate from the isolated atoms. Accordingly, the molecular density at each point for the individual atoms within the molecule is allocated in proportion to their respective contributions to the promolecule density at that point. The promolecular density is a direct sum of free contributions from all constituent atoms of the molecule. This charge analysis is shown to accurately describe the charge transfer reactions in water clusters,^48,49^ providing a well-suited approach for defining similar reactions in our system of interest.

*Water Cluster Calculations.* To better understand the mechanisms of Cl_2_ formation during the interaction HOCl with Cl^−^ ion at water surface, the reaction HOCl + Cl^–^ → Cl_2_ + OH^–^ in the presence of 6 water molecules was simulated. Ab initio quantum-chemical methods were applied such as the calculations of potential energy surfaces (PESs), determinations of transition states (TSs), and intrinsic reaction coordinate procedure (IRC). The PESs were initially studied using the PBE0 hybrid functional^50^ and 6-31+G* basis set using the Q-Chem^51^ and GAMESS^52^ programs. It has been shown that the PBE0 functional works well for water clusters and ion–water systems.^53^ Additionally, the DFT-D2 dispersion correction from Grimme is used.^54^ Energy for obtained structures were recalculated with MP2/6-311++G** level of theory. The number of negative eigenvalues of the Hessian matrix was examined for all of the stationary points. Additionally, zero-point energies, enthalpies, and Gibbs free energies (at 298 K) were used for thermochemical analysis. All resulting TSs were linked to their corresponding PES minima by descending along the reaction coordinate using the Gonzalez–Schlegel algorithm (IRC).^55^

## Supporting Information Available

Additional data showing geometric and electronic properties along trajectories, Figures S1–S8 and Tables S1 and S2 (PDF)
Transparent Peer Review report available (PDF)
