# Supplementary material for: Formation of Chlorine in the Atmosphere by Reaction of Hypochlorous Acid with Seawater
Source: J Phys Chem Lett. 2024 Jan 8;15(2):432–8. doi: 10.1021/acs.jpclett.3c03035 (PMC11139381; doi:10.1021/acs.jpclett.3c03035)

Supporting Information

Formation of Chlorine in the Atmosphere by Reaction of Hypochlorous Acid with Seawater

*Imon Mandal,[1] Natalia V. Karimova,[2] Itai Zakai,[1] R. Benny Gerber[1,2]**

[1]The Fritz Haber Center for Molecular Dynamics, Institute of Chemistry, The Hebrew University of Jerusalem, Jerusalem 91904, Israel

[2]Department of Chemistry, University of California, Irvine, California 92697, USA

***Corresponding Author**

Email for correspondence: robertbenny.gerber@mail.huji.ac.il

Author ORCIDs

Imon Mandal: 0000-0001-9680-8407

Natalia V. Karimova: 0000-0002-4616-1884

Itai Zakai: 0000-0002-0543-6562

R. Benny Gerber: 0000-0001-8468-0258

Table S1. Calculated (B3LYP/def2-TZVPD, gas phase) parameters for the (HOCl)…$(Cl^{-})_{aq}$ complexes reported in this study. *R* is the bond length (Å), and ∠ is the bond angle.

Geometric parameters	Hydrogen bonded (HOCl)…$(Cl^{-})_{aq}$	Halogen bonded (HOCl)…$(Cl^{-})_{aq}$
R(Cl^{-}-H/Cl) (Å)	1.90	2.53
R(Cl-O) (Å)	1.69	1.87
R(O-H) (Å)	1.03	0.97
∠HOCl	105.3º	100.2º
∠Cl^{-}···H/Cl-O	175.2º	179.6º

Figure S1. RMSD of the halogen bonded (HOCl)…$(Cl^{-})_{aq}$ along the time trajectories for all 10 simulations. Different colors indicate simulations with different velocities (see Method).

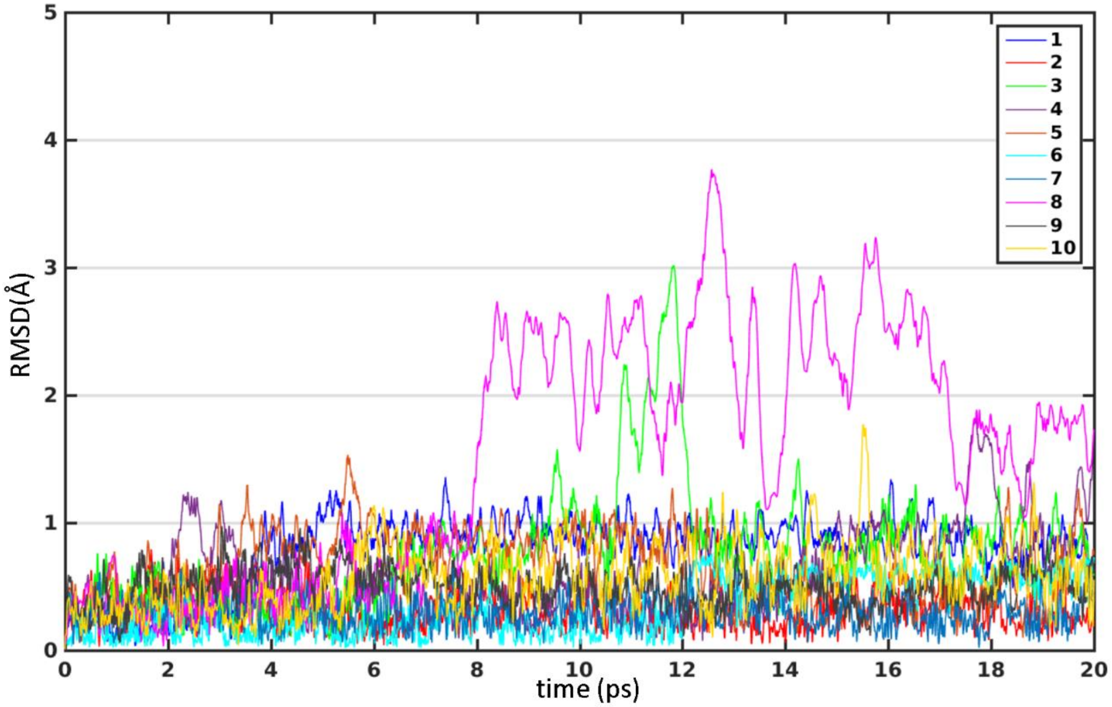

Figure S2. RMSD of the hydrogen bonded (HOCl)…(Cl^-)$_{aq}$ along the time trajectories for all 5 simulations. Different colors indicate simulations with different velocities (see Method). Snapshots of the hydrogen bonded (HOCl)…(Cl^-)$_{aq}$ complexes at time 10 ps and 20 ps for representative simulation are shown in the inset.

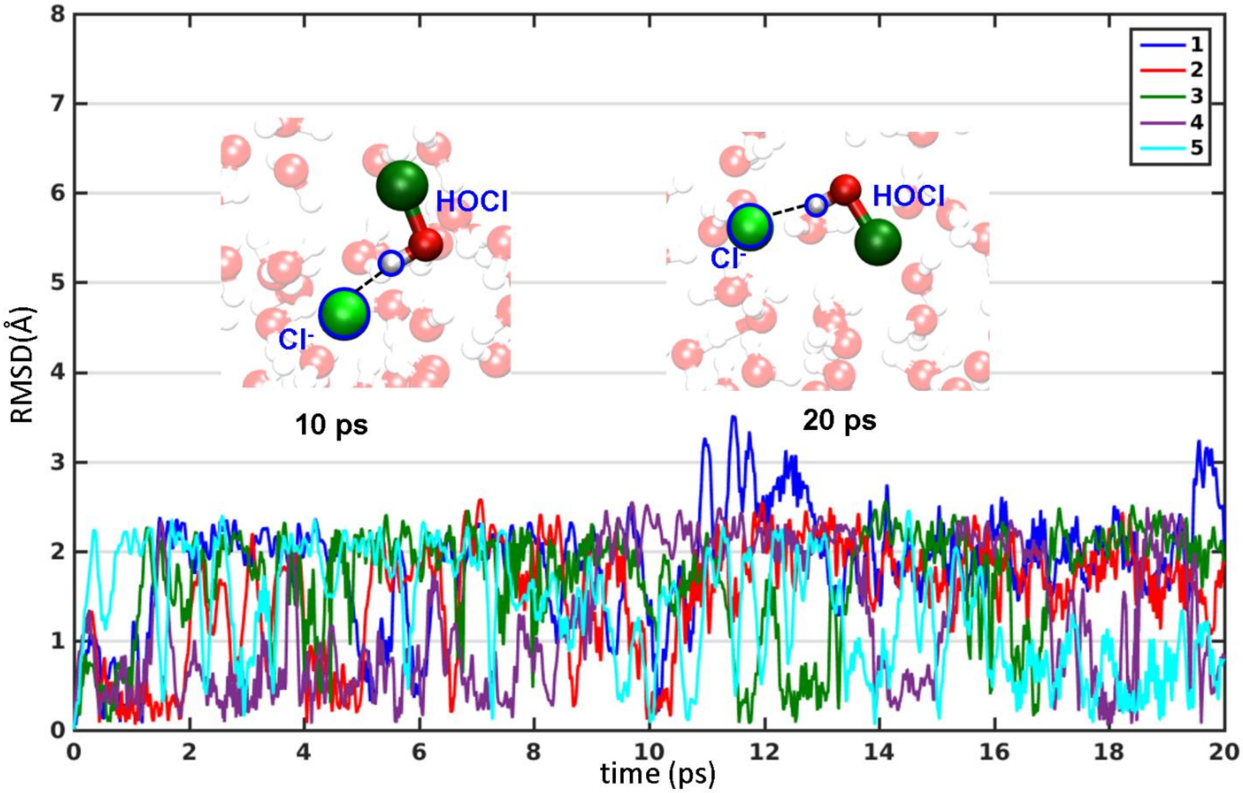

Figure S3. Bond lengths of the hydrogen bonded (HOCl)…(Cl-)$_{aq}$ along the time trajectories for all 5 simulations. Different colors indicate simulations with different velocities (see Method).

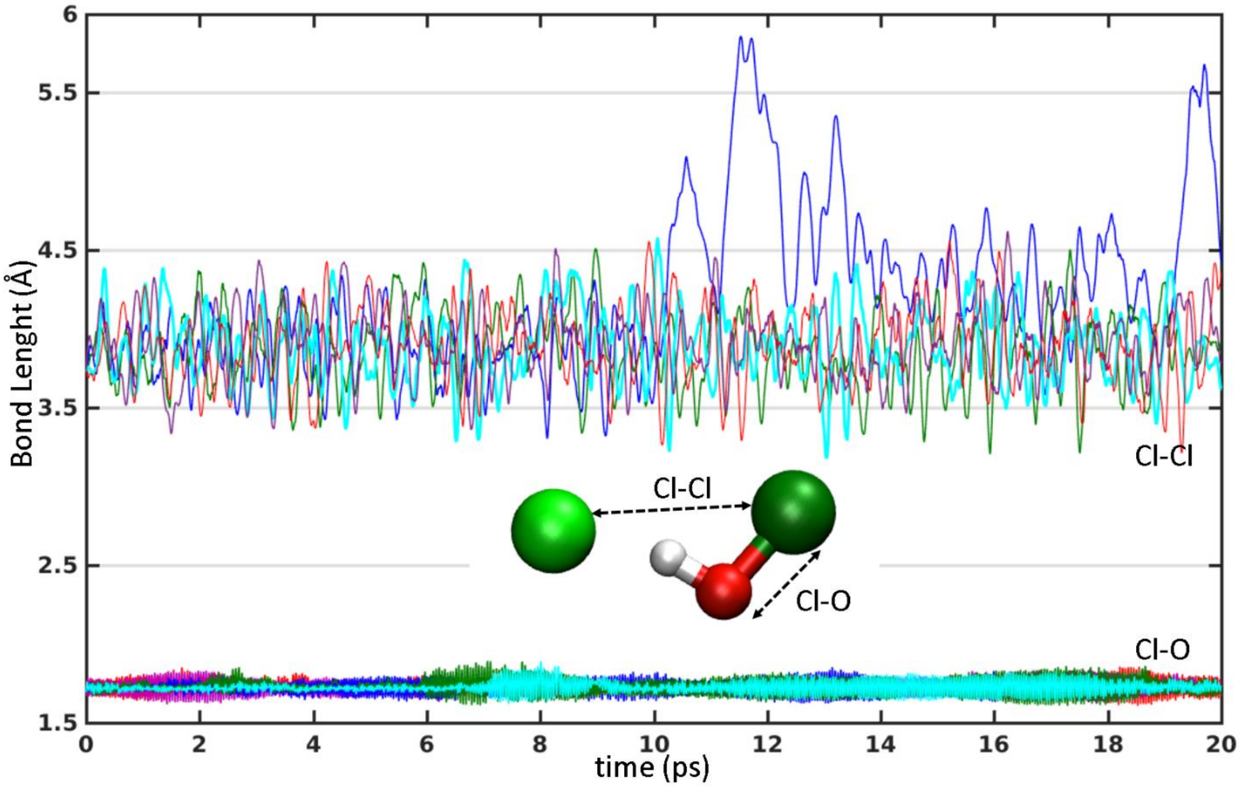

Figure S4. Bond lengths and partial charges along 2 transiently reacting trajectories at neutral pH. a) and b) Time evolution of the bond lengths (Cl-Cl^- and Cl-O) and Hirshfeld partial charges (Cl^-, Cl and summation of O and H from HOCl) of halogen bonded (HOCl)…$(Cl^-)_{aq}$ complexes along trajectory. The black dotted lines in a) and b) are eye guides for the time of the transient Cl_2 formation. Another trajectory is shown in Figure 2.

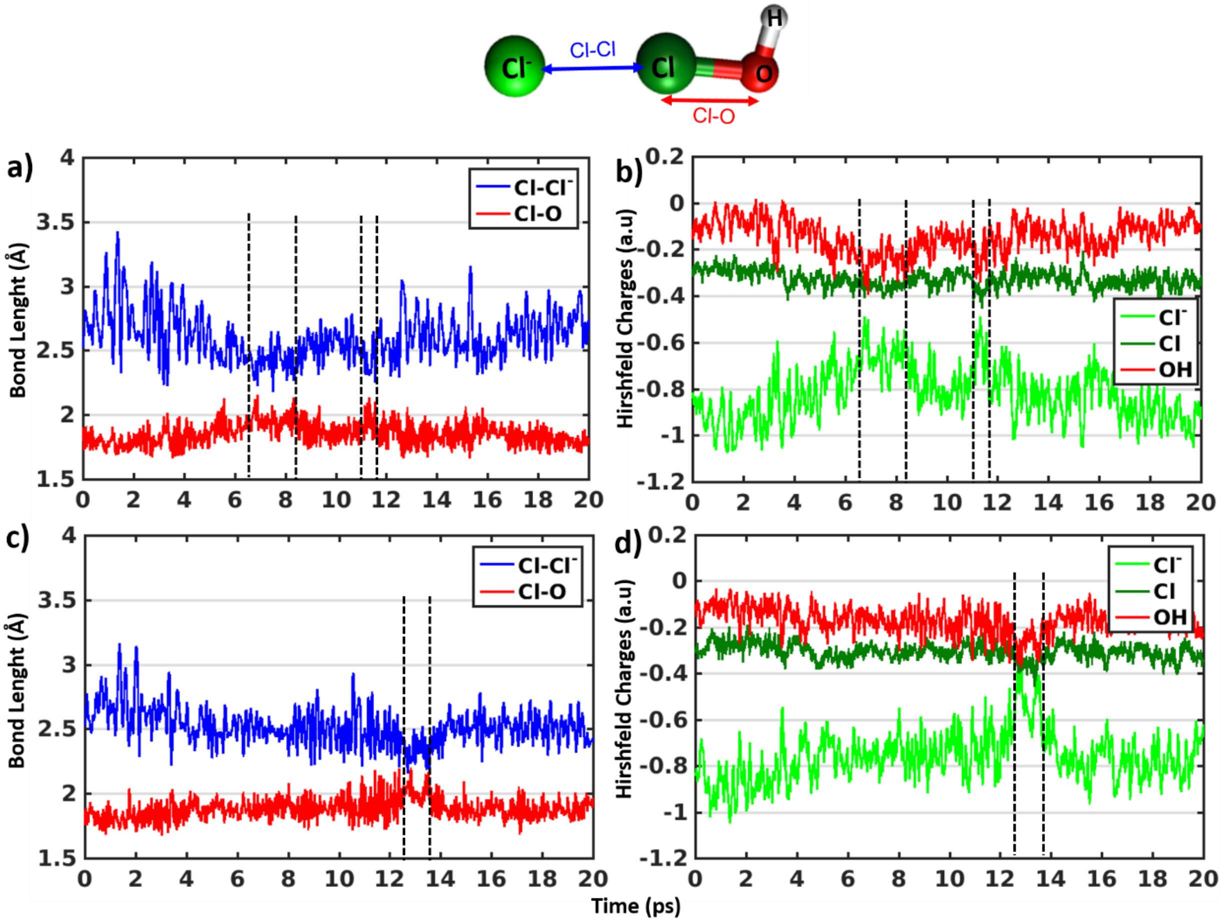

Figure S5. Pathways of reaction Cl_2 formation in the system (HOCl)…$(Cl^-)_{aq}/(H_2O)_6$, gas phase calculations (MP2/6-311++G**//PBE0-D/6-31+G*). Blue arrows show the proton migration from water molecules to OH^- fragment of HOCl.

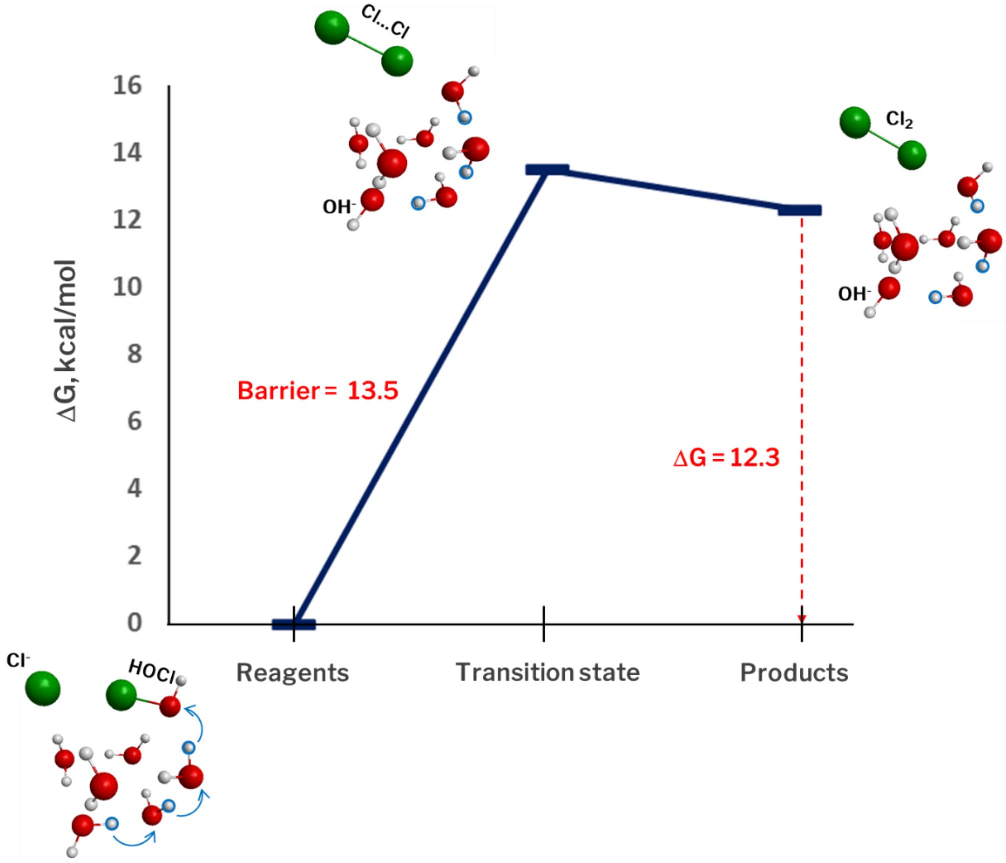

Figure S6. Bond lengths and partial charges for whole trajectory for Figure 3a) and b) Time evolution of the bond lengths (Cl-Cl$^-$, Cl-O and O-H) and Hirshfeld partial charges (Cl$^-$, Cl and summation of O and H from HOCl) of halogen bonded (HOCl)…(Cl$^-$)$_{aq}$ complexes along ~23 ps trajectory.

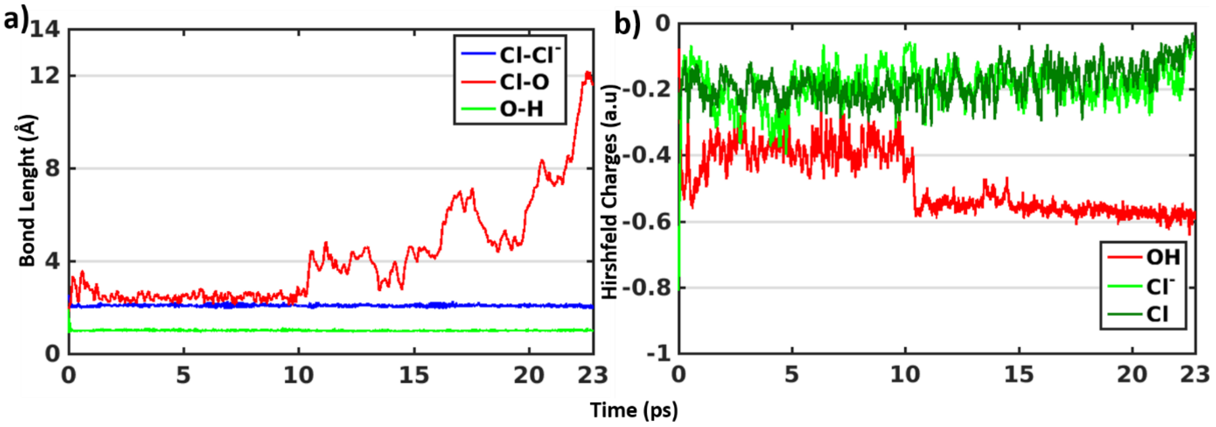

Table S2 Geometric parameters (r and θ defined in the main text) for 4 different simulations with different orientations of hydronium ions with respect to HOCl.

Geometric parameters	r(Å)	θ(degree)
Sim 1	1.91	109.38
Sim 2	1.89	109.82
Sim 3	1.83	97.60
Sim 4	1.83	81.72

Figure S7. Bond lengths and partial charges along 3 reacting trajectories at acidic pH a), c) and e) Time evolution of the bond lengths (Cl-Cl$^-$, Cl-O and O-H) and b), d) and f) Hirshfeld partial charges (Cl$^-$, Cl and summation of O and H from HOCl) of halogen bonded (HOCl)…$(Cl^-)_{aq}$ complexes along Sim 2-4 trajectories mentioned in Table S2. The black dotted lines are eye guides for the final time of the non-reversible Cl_2 formation at acidic pH. Similar data for Sim 1 is provided in Figure 3. Although Sim 1-4 are ~20 ps long simulations, data are presented for the first few 100 ps to effectively illustrate the changes in bond lengths and Hirshfeld partial changes during Cl_2 formation.

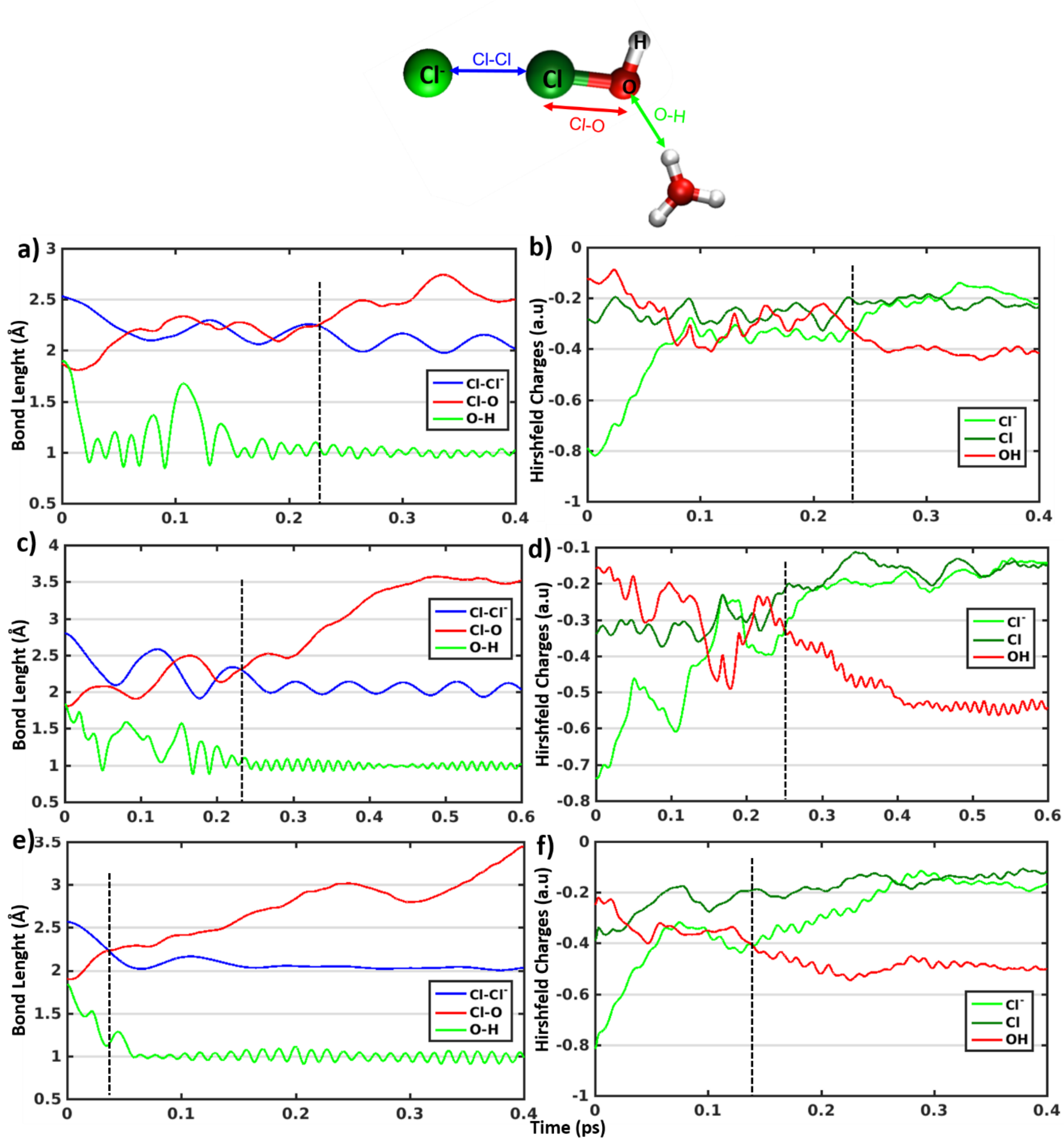

Figure S8. a) and b) Time evolution of the distance between geometric center of the water slab and the formed Cl_2 and Hirshfeld partial charge (summation of Cl^- and Cl from HOCl) of halogen bonded (HOCl)…$(Cl^-)_{aq}$ complexes along Sim 4 trajectory. Similar data for Sim 1 is presented in Figure 4.

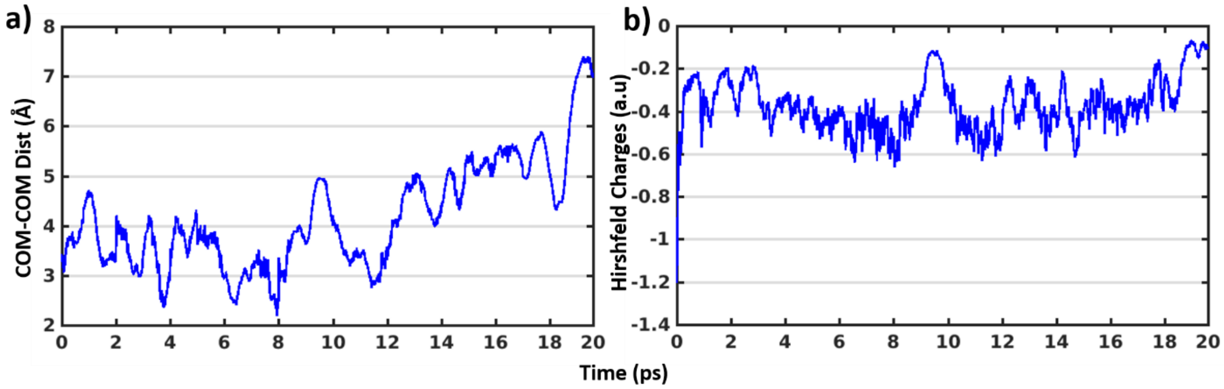

Supplement: Supplementary file 1 — jz3c03035_si_001.pdf [file jz3c03035_si_001.pdf]
