# Supplementary material for: Formation of Chlorine in the Atmosphere by Reaction of Hypochlorous Acid with Seawater
Source: J Phys Chem Lett. 2024 Jan 8;15(2):432–8. doi: 10.1021/acs.jpclett.3c03035 (PMC11139381; doi:10.1021/acs.jpclett.3c03035)
Supplement: Supplementary file 2 — jz3c03035_si_002.pdf [file jz3c03035_si_002.pdf]

Responses to the reviewers and revisions made

Dear Editor,

We thank you for conveying to us the comments of the reviewers. We thank the reviewers for their thoughtful and constructive comments. We are also grateful to the reviewers for their encouraging views on the manuscript. We revised the paper to address all the concerns of the reviewers. We believe that this has indeed improved the paper. Our responses to the comments of the reviewers and the revisions made accordingly are as follows:

Reviewer: 1

Summary:
This contribution is a nice computational study of the formation of the chlorine molecule on the seawater surface. The key reaction is between hypochlorous acid and chlorine anion on the surface, where water molecules surround the transition state complex. A key finding is such that water has to be slightly acid, i.e., protons are required for the production of Cl2.
Ab initio molecular dynamics is used to obtain the results presented. It is hard to see that more advanced theories could be used for this rather complicated problem.
I like that the authors have worked with this important problem. They have obtained interesting results which have wider significance considering atmospheric chemistry.

Response: We thank the reviewer for his/her encouraging remarks.

Comment 1: The authors state that the seawater pH value is about 8. They should elaborate a bit whether this can be considered to be an acidic value concerning the main finding of this contribution.

Authors reply and revisions made: We appreciate the reviewer's concern. In the manuscript we have stated that "Sea salt aerosols originate at the same pH as seawater (~8) but, within minutes, undergo a pH drop of a few pH units" which indicated that seawater pH is alkaline. However, there are many conditions where locally sea water is acidic e.g., the aerosol generated from the sea water is acidic. Moreover, recent reports show substantial fraction of emitted CO_2 emissions absorption by the oceans is resulting in a reduction of seawater pH[1,2] which would lead to reduce the seawater pH towards acidic, leading interfacial Cl_2 formation. In the revised manuscript, we added the sentence "However, the value of pH ~8 is an average value and there are many conditions where locally sea water is acidic" to clarify the reviewer's concern (highlighted in yellow).

Comment 2: It is mentioned that all H atoms have been substituted by D atoms at the crucial part of the simulation to suppress quantum mechanical tunnelling. First, this should also be said clearly in the main text. Second, this idea does not help with the

quantum mechanical zero-point energy which can be different for reactants and products. This could be elaborated a bit in the main text.

Authors reply and revisions made: We thank the reviewer to point out the valuable point. In the revised version of the manuscript, we have added "We substitute hydrogen atoms by deuterium to accommodate a larger time step for the simulations" (highlighted in yellow) in the Section "*Stability of (HOCl)…(Cl⁻)$_{aq}$ complexes in water*". We agree also with the second point of the reviewer, about the zero-point energy difference of the reactants and products. However, at 300K (temperature at which we simulated our systems) the role of zero-point energy is expected to be small. Therefore, we neglected the effect in the present study.

Minor points:

Response: We are grateful to the reviewer to carefully spot the typographical issues.
1) All non SI-units (Å, kcal/mol, Ry) should be defined as SI units first time these appear in the manuscript.

Authors reply and revisions made: We added 1 Å = 1×10^{-10} m in Page 6, 1 kcal/mol = 4.184 kJ/mol on Page 7 and 1 Ry = 1.097×10^{7} m^{-1} on Page 14.

2) a H-Cl- -> an H-Cl- and an Cl-Cl- -> a Cl-Cl-

Authors reply and revisions made: We change a $H\text{-}Cl^-$ → an $H\text{-}Cl^-$ and an $Cl\text{-}Cl^-$ → a $Cl\text{-}Cl^-$ on Page 6.

3) Data is -> Data are (Fig.3.)

Authors reply and revisions made: We modified Data is to Data are in Figure 3.

Reviewer: 2

Summary:
The activation (oxidation) of halides to reactive forms is of great importance in atmospheric chemistry. In spite of many experimental studies, both in the lab and in the field, real questions remain concerning the molecular mechanisms involved. In this work the authors describe ab-initio MD studies of the reaction of HOCl with Cl^- at (or near to) an air-water interface. They present arguments that the reaction proceeds via a halogen-bonded complex, and that the presence of a hydronium ion in the solvation shell promotes reaction significantly.

Response: We thank the reviewer for his/her encouraging remarks.

Comment 1: While both of these findings are really interesting the really novel aspect of the work to me is the existence of a long-lived halogen-bonded complex, and its

importance to the reaction. That this is non-intuitive is hinted by the authors, but the physical reason(s) are not addressed. Indeed, the authors statte that the halogen-bonded complex is less strongly bound than the hydrogen bonded one, yet both are (equally) long-lived!! Please - some physical insights here about how this happens! I assume that solvation plays some role; understanding exactly what role will surely give greater insight into this reaction.

Authors reply and revisions made: We regret the lack of clarity about the physical insights of the stability of halogen bonded complexes. As the reviewer pointed out correctly, both hydrogen and halogen bonded complexes are (equally) long lived during our simulation time of 20 ps. We clarify that these complexes are stable at least 20 ps which is enough to act as pre-reactive complexes. The exact lifetimes of these complexes are not known as we run simulation only up to 20 ps, longer simulations would be costly due to system size and not relevant of the problem addressed in the paper. The reason of stabilization of halogen bonded complexes in water is the water envelop surrounding the complexes provides additional binding stabilization. In the case of hydrogen bonded complexes, the hydrogen bond partner of HOCl has competition between Cl^- ion and other solvent water molecules. We added in the revised manuscript the sentence "This stabilized water envelop provides enough lifetime to the halogen bonded complexes" (highlighted in yellow) on Page 6 to clarify this point.

Comment 2: I also wonder about the authors' thoughts on the role of entropy in driving the reaction. The acid-promoted reaction has water as a product; in solution this would not necessarily be favored to the same extent as at an interface. So the mechanism *may* be somewhat interface-specific - do the authors have thoughts on this?

Authors reply and revisions made: We agree with the reviewer's comment. In fact, because of the entropy effect, the reaction is expected to be enhanced at the interface with respect to bulk. However, we did not quantify the effect. We have referred about this in the on Page 12 (highlighted in yellow). Moreover, we tried to put the complex inside the water slab (Data not shown), but after 3 ps the complex reaches the interface mentioned in the Computational Methods Section. As the pre-reactive complexes are only stabilized on the interface, the mechanism is interface specific.

Response to the Editor's comments:

We modified References 1-55 in the main text (highlighted in yellow) according to the JPCL format in the revised manuscript. We included the page numbers in the SI.
References:

(1) Ríos, A. F.; Resplandy, L.; García-Ibáñez, M. I.; Fajar, N. M.; Velo, A.; Padin, X. A.; Wanninkhof, R.; Steinfeldt, R.; Rosón, G.; and Pérez, F. F. Decadal acidification in the water masses of the Atlantic Ocean. *Proc. Natl. Acad. Sci. U.S.A.* **2015,** *112,* 9950-9955.

(2) Dore, J. E.; Lukas, R.; Sadler, D. W.; Church, M. J.; and Karl, D. M. Physical and biogeochemical modulation of ocean acidification in the central North Pacific. *Proc. Natl. Acad. Sci. U.S.A.* **2009,** *106,* 12235-12240.
